# Effects of Sulfated Fucans from *Laminaria hyperborea* Regarding VEGF Secretion, Cell Viability, and Oxidative Stress and Correlation with Molecular Weight

**DOI:** 10.3390/md17100548

**Published:** 2019-09-25

**Authors:** Philipp Dörschmann, Georg Kopplin, Johann Roider, Alexa Klettner

**Affiliations:** 1 Department of Ophthalmology, University Medical Center, University of Kiel, Arnold-Heller-Str. 3, Haus 25, 24105 Kiel, Germany; Johann.Roider@uksh.de (J.R.); Alexa.Klettner@uksh.de (A.K.); 2 Alginor ASA, Haraldsgata 162, 5525 Haugesund, Norway; Georg@alginor.no; 3 Norwegian Biopolymer Laboratory (NOBIPOL), Department of Biotechnology and Food Science, NTNU, 7491 Trondheim, Norway

**Keywords:** fucoidan, fucan, age-related macular degeneration, VEGF, oxidative stress, *Laminaria hyperborea*, brown seaweed extracts, proliferation, molecular weight, retinal pigment epithelium

## Abstract

Background: Sulfated fucans show interesting effects in the treatment of ocular diseases (e.g., age-related macular degeneration), depending on their chemical structure. Here, we compared three purified sulfated fucans from *Laminaria hyperborea* (LH) regarding cell viability, oxidative stress protection, and vascular endothelial growth factor (VEGF) secretion in ocular cells. Methods: High-molecular-weight sulfated fucan (M_w_ = 1548.6 kDa, Fuc1) was extracted with warm water and purified through ultrafiltration. Lower-molecular-weight samples (M_w_ = 499 kDa, Fuc2; 26.9 kDa, Fuc3) were obtained by mild acid hydrolysis of ultrapurified sulfated fucan and analyzed (SEC-MALS (Size-exclusion chromatography-Multi-Angle Light Scattering), ICP-MS, and GC). Concentrations between 1 and 100 µg/mL were tested. Cell viability was measured after 24 h (uveal melanoma cell line (OMM-1), retinal pigment epithelium (RPE) cell line ARPE-19, primary RPE cells) via MTT/MTS (3-(4,5-dimethylthiazol-2-yl)-2,5-diphenyltetrazolium bromide/3-(4,5-Dimethylthiazol-2-yl)-5-(3-carboxymethoxyphenyl)-2-(4-sulfophenyl)-2H-tetrazolium) assay. Oxidative stress protection was determined after 24 h (OMM-1, ARPE-19). VEGF secretion was analyzed via ELISA after three days (ARPE-19, RPE). Results: Fuc2 and Fuc3 were antiproliferative for OMM-1, but not for ARPE. Fuc1 protected OMM-1. VEGF secretion was lowered with all fucans except Fuc3 in ARPE-19 and RPE. The results suggest a correlation between molecular weight and biological activity, with efficiency increasing with size. Conclusion: The LH sulfated fucan Fuc1 showed promising results regarding VEGF inhibition and protection, encouraging further medical research.

## 1. Introduction

*Laminaria hyperborea* (LH), commonly known as tangle or cuvie, belongs to the large brown seaweed family of the Laminariaceae (alias kelp). It mainly grows in the northeast Atlantic Ocean, especially around Scandinavia. LH, like all brown algal species, contains fucose-containing sulfated polysaccharides (FCSP), commonly known as fucoidan, as a cell wall component. FCSP predominantly based on sulfated L-fucose residues are defined as sulfated fucans [1]. FCSP are a very heterogeneous group of polysaccharides with strong variations in sugar composition, degree of branching and sulfation, and molecular weight. Their structure depends on different aspects, like species, harvest time, place, and extraction method [2]. This heterogeneity in structures also leads to a variety of different biological activities. Sulfated fucans have been described as being able to reduce oxidative stress and inflammation, as well as being capable of interfering with vascular endothelial growth factor (VEGF) and blood lipids [3]. These properties render sulfated fucans very interesting for medical purposes, especially for the treatment of age-related macular degeneration (AMD) [4]. AMD is the main cause of blindness in the industrialized world and may cause an irreversible loss in central vision in the elderly due to the degeneration of photoreceptors within the macula lutea. This may happen in two ways: starting in the early phase with the deposition of drusen and an accumulation of lipid peroxidation products (lipofuscin), it may progress to dry AMD, where retinal pigment epithelium (RPE) degeneration is followed by a death of photoreceptors, leading to geographical atrophy of the retina as a late stage [5,6]. In wet AMD, the major growth factor of blood vessels, VEGF, is abnormally increased, leading to the growth of blood vessels under and into the retina. Followed by edema and sometimes bleeding, it leads to a disruption of the retina [5]. The pathology of AMD is based on four molecular mechanisms that increase the risk of this illness: inflammation, complement activation, oxidative burden, and disturbed VEGF generation [5,7,8,9]. The only therapy possibility is repeated anti-VEGF injections into the eyes of the patient [10], which slows down the pathology of wet AMD. 

Because of their biological activities, FCSP are very interesting as possible new therapeutics for the treatment of AMD [3]. We showed previously that commercial sulfated fucan from *Fucus vesiculosus* reduces VEGF and the angiogenesis of RPE cells [4]. We also described the protective effects of *Saccharina latissima* (SL) sulfated fucan for ARPE-19 cells against oxidative stress insult; this fucan also lowered VEGF in ARPE-19 and RPE cells [11]. Other extracts from *Fucus serratus*, *Fucus distichus* subsp. *evanescens*, and *Laminaria digitata* also inhibited VEGF secretion in ARPE-19 cells [11]. The chemical structure and composition of sulfated fucan from LH has been previously described [12]. It was shown that the biological activity of LH sulfated fucan is dependent on the molecular weight and the degree of sulfation [12]. LH sulfated fucans with higher molecular weight and degree of sulfation were capable to reduce coagulation and had anti-inflammatory effects [12]. Stefaniak-Vidarsson et al. (2017) described the anti-proliferative effects of *Laminaria* sulfated fucans in activated human macrophages and showed that these sulfated fucans triggered tumor necrosis factor-α and interleukins 6 and 10 [13]. However, only few reports about the activity of LH sulfated fucans can be found in the literature. Because of the described connected effects to molecular weight and to the high degree of sulfation, as well as the different species-dependent structures and activities, we tested LH sulfated fucans additionally to the other mentioned species to find the best sulfated fucans for further AMD-relevant research.

With this study we wanted to investigate whether these LH sulfated fucans display toxic effects and whether they are capable of interfering with VEGF secretion and the oxidative burden in ocular cells, with a view to making an important step further to new AMD treatment possibilities. For this purpose, three pure sulfated fucans from LH were tested; these fucans differed only in their molecular weight and were harvested at the same time, at the same place, with the same extraction method to achieve high comparability.

## 2. Results

### 2.1. Structural Characterization of the Sulfated Fucans 

Monosaccharide anion-exchange chromatography of Fuc1 revealed a sugar composition of 97.0% ± 0.1% fucose (retention time 3.134 min) and 3.0% ± 0.1% galactose (retention time 7.910 min). Neither uronic acid residues nor glucose were detected. 

Mass spectrometry of Fuc1 revealed a sulfite content (-SO_3_) of 29.44%, which converts into a sulfation degree (DS) of approximately 1.7 sulfate groups per sugar residue (Table 1). Both (sugar composition and DS) are in full agreement with the previously elucidated molecular structure [12]. An overlay of the Raman spectra from samples Fuc1, Fuc2, and Fuc3 showed no change in the relative intensities of the sulfate group vibrations (822.5 cm^−1^, 839.8 cm^−1^, 1065.3 cm^−1^, 1262.4 cm^−1^) and methyl group vibrations (1340.9 cm^−1^, 1452.5 cm^−1^), indicating an equal ratio between the two groups and thereby showing that no desulfation occurred during the mild acid hydrolysis.

**Figure 1:**
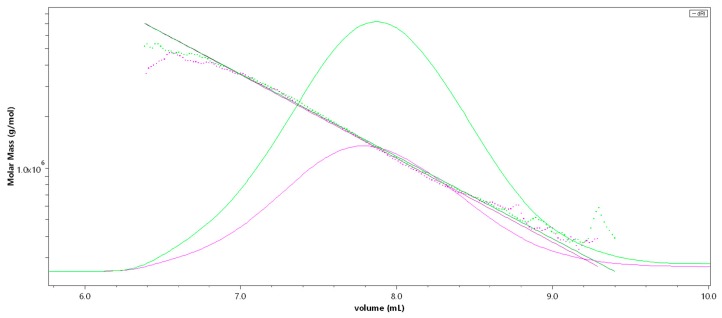
SEC-MALS (Size-exclusion chromatography-Multi-Angle Light Scattering) chromatogram of the high-molecular-weight sulfated fucan (Fuc1) giving M [g/mol] versus V [mL]; 50 µL injection (pink), 100 µL injection (green). The molar mass is plotted as a dotted line, the refractive index is displayed as an overlay.

**Figure 2:**
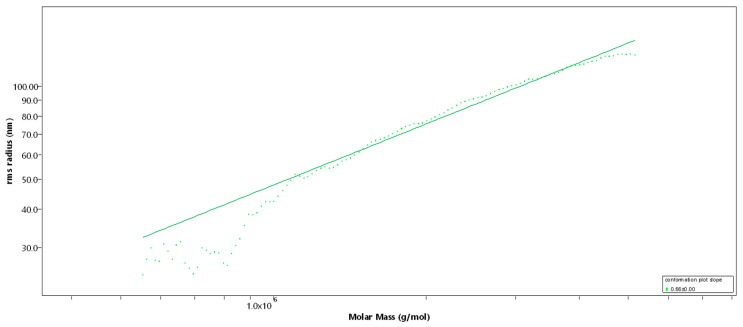
Rms conformation plot of the high-molecular-weight sulfated fucan (Fuc1) giving the rms radius [nm] versus M [g/mol], 100 µL injection. The slope (b) = 0.66 indicates a random coil.

SEC-MALS (Size-exclusion chromatography-Multi-Angle Light Scattering) analysis of Fuc1 revealed a very high molecular weight average of M_w_ = 1548 (± 4.1) kDa [14], resulting in an average degree of polymerization (DP_n_) of 3512, with an approximated average monosaccharide unit weight of 290 g/mol [12]. The molar mass was broadly distributed between 300 kDa and 7 MDa, displaying coherent results throughout different injected volumes (50 µL and 100 µL, c = 1 g/L, see Figure 1). The radius of gyration as the Z-average (R_z_) was found to be 83.0 nm. The overall shape of the molecule was determined through an rms conformation plot (rms radius [nm] versus M [g/mol], see Figure 2), displaying a slope (b) of 0.66 and thus indicating an overall random coil shape of the molecule (sphere b = 0.3; random coil b = 0.5; rigid rod b = 1) [15]. Considering the previously reported high degree of branching of the molecule, while having obtained the same sugar composition and degree of sulfation as shown in Kopplin et al. (2018) [12], the obtained shape of a random coil for an LH sulfated fucan molecule 3 times bigger supports the previously suggested overall structure of a large, highly flexible main chain with short side chains. Fuc2 and Fuc3 showed almost identical structural features, only differing in their degree of polymerization. 

### 2.2. Effects on Cell Viability

**Figure 3:**
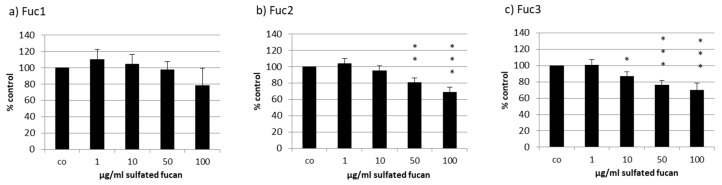
Cell viability was tested in uveal melanoma cell line OMM-1 after treatment with *Laminaria hyperborea* (LH) sulfated fucans Fuc1 (**a**), Fuc2 (**b**), and Fuc3 (**c**) for 24 h. Cell viability was determined via MTS (3-(4,5-Dimethylthiazol-2-yl)-5-(3-carboxymethoxyphenyl)-2-(4-sulfophenyl)-2H-tetrazolium) assay and is shown as the mean and standard deviation in relation to a 100% control. Significance was evaluated with ANOVA; * *p* < 0.05, ** *p* < 0.01, *** *p* < 0.001 compared to control (*n* = 4).

The uveal melanoma cell line OMM-1 was treated with the three LH sulfated fucans for 24 h; after that, an MTS (3-(4,5-Dimethylthiazol-2-yl)-5-(3-carboxymethoxyphenyl)-2-(4-sulfophenyl)-2H-tetrazolium) assay was conducted. In all three cases a dose-dependent decrease in cell viability starting with 1 µg/mL could be seen (Figure 3), but only Fuc2 and Fuc3 showed significant effects: 50 and 100 µg/mL Fuc2 lowered cell viability to 83% ± 5% (*p* < 0.01) and 76% ± 6% (*p* < 0.001), respectively. Quantities of 10, 50, and 100 µg/mL Fuc3 reduced cell viability even further to 87% ± 6%, 76% ± 5%, and 70% ± 9%, respectively. Fuc1, the sulfated fucan with the highest molecular weight, showed no significant effects on cell viability but showed a tendency at 100 µg/mL, which is not significant because of the higher variability.

**Figure 4:**
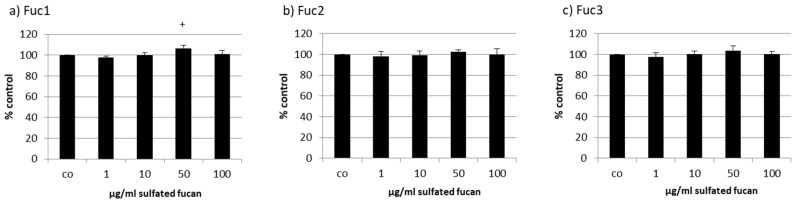
Cell viability was tested in retinal pigment epithelium (RPE) cell line ARPE-19 after treatment with LH sulfated fucans Fuc1 (**a**), Fuc2 (**b**), and Fuc3 (**c**) for 24 h. Cell viability was determined via MTS assay and is shown as the mean and standard deviation in relation to a 100% control. Significance was evaluated with ANOVA compared to the control (*n* = 4); ^+^ *p* < 0.05.

The human RPE cell line ARPE-19 was treated with the three LH sulfated fucans for 24 h and tested with a consecutive MTS assay. Only 50 µg/mL Fuc1 increased cell viability slightly (Figure 4).

**Figure 5:**
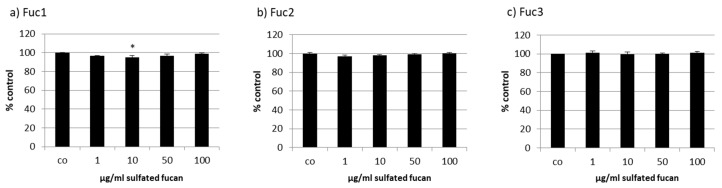
Cell viability was tested in RPE cells after treatment with LH sulfated fucans Fuc1 (**a**), Fuc2 (**b**), and Fuc3 (**c**) for 24 h. Cell viability was determined via MTT (3-(4,5-dimethylthiazol-2-yl)-2,5-diphenyltetrazolium bromide) assay and is shown as the mean and standard deviation in relation to an untreated control (100%). Significance was evaluated with ANOVA compared to the control (*n* = 3); * *p* < 0.05.

Primary porcine RPE cells were treated with the three LH sulfated fucans for 24 h and tested with an MTT (3-(4,5-dimethylthiazol-2-yl)-2,5-diphenyltetrazolium bromide) assay. The 10 µg/mL Fuc1 lowered viability slightly, but not to a biologically relevant degree (Figure 5). This paved the way for extended incubation times to measure VEGF secretion after 72 h (see below).

### 2.3. Oxidative Stress Protection

Oxidative stress protection by sulfated fucans from brown seaweed was already shown before for OMM-1 with *Fucus vesiculosus* sulfated fucan from Sigma-Aldrich [16] and with sulfated fucans from other seaweed, especially from SL, which was also protective for ARPE-19 [11]. So, these two cellular model systems are suitable for testing LH sulfated fucans.

**Figure 6:**
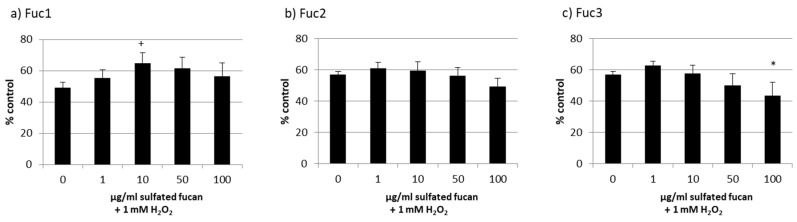
OMM-1 cell viability after treatment with Fuc1 (**a**), Fuc2 (**b**), and Fuc3 (**c**) and stress insult after 30 min incubation with the sulfated fucans. The oxidative agent was 1 mM H_2_O_2_, which reduced cell viability to under 60% in all cases. Viability was tested via MTS assay and is shown as the mean and standard deviation in relation to an untreated control (100%). The 10 µg/mL Fuc1 showed significant protective effects for the cell line. Significance was evaluated via ANOVA; */^+^ *p* < 0.05, versus 0 µg/mL sulfated fucan + 1 mM H_2_O_2_ (*n* = 4).

Starting with the melanoma cell line OMM-1, stress induction with 1 mM H_2_O_2_ led to decreased cell viability of between 49% and 57% (Figure 6). The addition of 1–100 µg/mL Fuc2 showed no protection. The same outcome was achieved via treatment with Fuc3, which significantly reduced viability at 100 µg/mL (44% ± 9%, *p* < 0.05). Also, 50 µg/mL Fuc3 showed a slightly antiproliferative effect which was not significant. Only the sulfated fucan with the highest molecular weight, Fuc1, had significant protective effects at 10 µg/mL (65% ± 7%, *p* < 0.05). 

RPE cells have the important role of protecting the cells of the retina against oxidative burden [7]. The RPE cell line ARPE19 was described to be rather resistant against hydrogen peroxide [17]. However, treatment with 0.5 mM *tert*-butyl hydroperoxide (TBHP) has a significant, consistent effect on the cell viability of ARPE-19 after 24 h, as previously shown [11].

**Figure 7:**
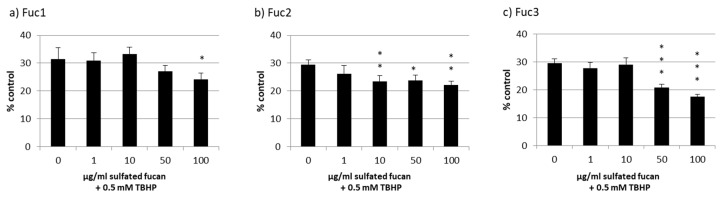
ARPE-19 cell viability after treatment with Fuc1 (**a**), Fuc2 (**b**), and Fuc3 (**c**) and stress insult after 30 min incubation with the sulfated fucans. The oxidative agent was 0.5 mM *tert*-butyl hydroperoxide (TBHP), which reduced cell viability to nearly 30% in all cases. Viability was tested via MTS assay and is shown as the mean and standard deviation in relation to an untreated control (100%). No sulfated fucan showed a significant protective effect for the RPE cell line. Significance was evaluated with ANOVA; * *p* < 0.05, ** *p* < 0.01, *** *p* < 0.001, versus 0 µg/mL sulfated fucan + 0.5 mM TBHP (*n* = 4).

Cell viability was effectively lowered to nearly 30% through oxidative stress with TBHP, but all LH sulfated fucans lowered the viability additionally to a lesser extent (Figure 7); the strongest effect was from 100 µg/mL of the Fuc3 sulfated fucan, which lowered the viability even further down to 18% ± 1% (*p* < 0.05).

### 2.4. VEGF Secretion

The effect of the three LH sulfated fucans on the secretion of VEGF was determined in the primary, porcine RPE cells and the human RPE cell line ARPE-19. ARPE-19 secreted nearly 4 times less VEGF compared to the primary RPE cells (ARPE-19: 66 pg/h and RPE: 243 pg/h). The VEGF content secreted into the supernatant of the cells was collected for ARPE-19 over 24 h and for RPE over 4 h because of the higher VEGF outcome. At this time point a medium exchange with added sulfated fucans was performed. The VEGF content [pg/mL] was normalized to the cell viability (after 72 h sulfated fucan treatment), giving the ratio of VEGF/cell viability (in arbitrary units [arb. unit]). The cell viability of both cell types with all sulfated fucans and all test concentrations was essentially unaffected (data not shown).

**Figure 8:**
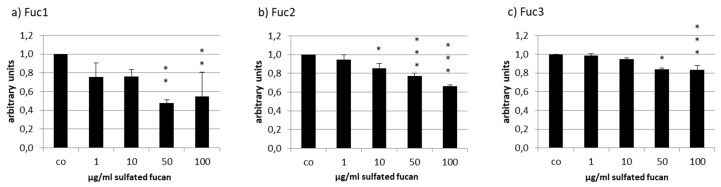
Vascular endothelial growth factor (VEGF) secretion of ARPE19 cells after incubation with Fuc1 (**a**), Fuc2 (**b**), and Fuc3 (**c**). VEGF content was analyzed with ELISA and normalized to cell viability. All LH extracts reduced VEGF content with 50 µg/mL Fuc1 as the most efficient. Significance was evaluated with ANOVA; * *p* < 0.05, ** *p* < 0.01, *** *p* < 0.001 compared to the control (*n* = 4).

The secreted VEGF was lowered by all tested sulfated fucans in ARPE-19 (Figure 8). Fuc3 reduced VEGF to 0.84 ± 0.05 [arb. unit], *p* < 0.001, and Fuc2 lowered it to 0.66 ± 0.01 [arb. unit], *p* < 0.001, at 100 µg/mL. The effect of Fuc2 and Fuc3 on VEGF was concentration dependent. This did not apply for Fuc1, the sulfated fucan with the highest molecular weight, which reduced VEGF to 0.48 ± 0.04 [arb. unit], *p* < 0.01, at 50 µg/mL and had the strongest effect of all sulfated fucans at this concentration.

**Figure 9:**
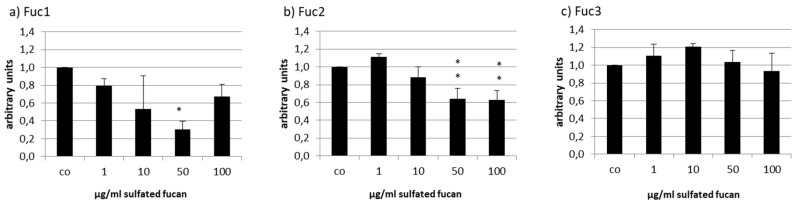
VEGF secretion of RPE cells after incubation with Fuc1 (**a**), Fuc2 (**b**), and Fuc3 (**c**). VEGF content was analyzed via ELISA and normalized to the cell viability. Fuc2 and Fuc1 reduced the VEGF content. Significance was evaluated via ANOVA; * *p* < 0.05, **p < 0.01, compared to the control (*n* = 3).

Due to the high amount of VEGF production in RPE cells, weaker effects could be expected, but again VEGF was reduced by Fuc2 and Fuc1 (Figure 9). Fuc3, the smallest LH sulfated fucan, seemed to increased VEGF secretion at 10 µg/mL (1.21 ± 0.04 [arb. unit]); however, this change was not significant. Fuc2 reduced VEGF at 50 µg/mL to 0.64 ± 0.12 and at 100 µg/mL to 0.63 ± 0.10 [arb. unit], *p* < 0.01, and Fuc1 reduced VEGF at 50 µg/mL to 0.30 ± 0.09 [arb. unit], *p* < 0.001. Once more, Fuc1 had the highest efficiency and, similar to what was seen in ARPE-19, again showed the strongest effect at 50 µg/mL.

## 3. Discussion

The different biological effects of sulfated fucans make them very interesting for medical research, but each sulfated fucan should be exactly characterized because of the high heterogeneity concerning these biological effects. Effects that are beneficial for the treatment of AMD were already described [2]. In this study we compared three purified sulfated fucans extracted from LH by Alginor ASA. The chemical structure was previously characterized and differs among the three samples only by the molecular weight average, which makes comparison in relation to the molecular weight possible. The aim was to prescreen these extracts as to whether they are suitable for further AMD-related research. To our knowledge, LH sulfated fucans had not been tested for this purpose before. Oxidative burden and VEGF secretion are two important factors in the risk of developing AMD and were therefore the main focus of this study. Because RPE cells are the target of the pathological mechanisms, they were chosen as the cellular models. The melanoma cell line OMM-1 acted as a model for oxidative stress protection as it is more susceptible to oxidative stress than the RPE cells. 

First, the LH sulfated fucans were tested for their effects on the viability of primary RPE cells and the RPE cell line ARPE-19. In both cases there were no significant effects, which is in line with earlier studies like that by Bittkau et al. 2019, which reported that sulfated fucans in general are not antiproliferative for adherent cell lines [18]. However, this is dependent on the specific fucoidan and model system. For example Banafa et al. described antiproliferative effects of *Fucus vesiculosus* sulfated fucan in human breast cancer cells [19]. It was demonstrated that it arrests the cell cycle at G_1_, has pro-apoptotic properties, and enhances ROS formation [19]. Fuc2 and Fuc3 showed cell viability lowering effects in the melanoma cell line OMM-1, which could be desirable for possible use in anticancer treatments. They both have a size below 500 kDa, so the effects could be connected to the molecular weight and structure. Possibly, these sulfated fucans interfere with fibronectin, disturbing the adherence of the tumor cell line [20]. Of note, the attachment of OMM-1 to well plates is rather weak and the cells have no tight junctions, in contrast to the RPE and ARPE-19 cell lines. In addition, OMM-1 cells were treated at subconfluence and the sulfated fucans could slow down the proliferation of this cell line, whereas the RPE cells were already confluent.

Because oxidative burden is considered one of the main causes of the development of AMD [7], the capability of the sulfated fucans to lower oxidative stress induced by TBHP or H_2_O_2_ was investigated. In ARPE-19, no protective effects could be detected, and 100 µg/mL Fuc3 had a cumulative toxic effect (also for OMM-1), which means that the smallest LH sulfated fucan enhanced the toxic effect of TBHP and H_2_O_2_. The same outcome was achieved for the OMM-1 cell line, except for the biggest sulfated fucan Fuc1, which was protective. These results are in contrast to those for the sulfated fucans of *Sargassum angustifolium*, in which the antioxidant activity through the scavenging of radicals and reducing power increased with decreasing size of the sulfated fucan (size range from 421 to 64 kDa) [21]. Because Fuc2 and Fuc3, which were also in that range, had no effect at all, and due to the absence of polyphenols by virtue of the high purity, it could be assumed that Fuc1 had no scavenging effect but rather interacted with the antioxidant defense system of the OMM-1 cell line. The RPE cells have an already improved antioxidant defense system, controlled via Nrf-2 (transcription factor nuclear factor erythroid 2-like 2), because of the high accumulation of oxygen radicals in the photoreceptors [7]. We already showed before that several sulfated fucans have protective effects in the OMM-1 cell line, but rarely do they in the ARPE-19 cell line [11,16]. OMM-1 cells have decreased superoxide dismutase activity [22], which could mean that the protective effects of ARPE-19 are concealed by already existing antioxidant enzyme activity. That sulfated fucans can activate superoxide dismutase and Nrf-2 was shown previously [23,24,25,26].

A further important factor, mainly for the development of wet AMD, is the secretion of VEGF, which supports the angiogenesis of blood vessels in the eye. ARPE-19 cell lines and the primary RPE cells were tested for their VEGF output after sulfated fucan treatment for three days. The secreted VEGF was normalized to the cell viability. As shown before, primary RPE cells produce significantly more VEGF compared to the cell line [11] (nearly 4 times more than ARPE-19); therefore, any significant reduction of the growth factor in primary RPE cells is even more remarkable. All three LH sulfated fucans decreased VEGF significantly at 50 and 100 µg/mL, with efficiency decreasing with smaller size in the RPE and ARPE-19 cells. This reducing effect could be due to the direct binding of VEGF, which inhibits the activation of the VEGF receptor. The negatively charged side chains of sulfated fucans can interact with the positively charged residues of the VEGF molecule, which could explain the interference in the binding between VEGF and its receptor [27]. This corresponds to findings in the literature [28,29]. It could be assumed that bigger size of the sulfated fucan is more efficient in VEGF inhibition. Also, direct interaction with the VEGF receptor (VEGF-R) [28] or the suppression of the VEGF and VEGF receptor genes could be possible, as VEGF has been shown to be involved in its own regulation [3,30,31,32,33]. Additional VEGF binding assays should be performed to determine the affinity of the fucoidan to the growth factor, which should be considered for further studies with LH sulfated fucans. Even more striking is the effect in the RPE cells, in which Fuc1 and Fuc2 also decreased VEGF despite the higher secretion. For both cell types, 50 µg/mL Fuc1 had the strongest effect. Fuc3 showed a tendency to stimulate VEGF secretion in RPE cells; however, this was not significant and its biological relevance is therefore uncertain. Nevertheless, it is of interest, as this small sulfated fucan may interact with VEGF differently to its high-molecular-weight counterpart, possibly by binding to VEGF in a VEGF-R-activating manner [34] The DS is also an important factor for the angiogenesis influencing effect [29], but the DS for all three sulfated fucans was the same.

The monosaccharide analysis by anion-exchange chromatography revealed a polysaccharide almost exclusively composed of fucose (97.0% fucose, 3.0% galactose). In addition, the sulfite content (-SO_3_) of 29.44% (DS = 1.7) determined by ICP-MS is in strong agreement with data from the previously elucidated LH sulfated fucan structure [12]. The obtained rms conformation plot slope (b) of 0.66 through SEC-MALS revealed a random coil shape for both Fuc1 and Fuc2, further supporting the previously suggested long backbone structure (1→3 glycosidic linkages) with short side chains at 1→2 and 1→4 branching points. Therefore, the main difference between Fuc1, Fuc2, and Fuc3 was the degree of polymerization, directly associating the effects on proliferation, oxidative stress protection, and VEGF secretion with one structural property. 

It has been shown that fucoidan, due to its high degree of sulfation, is able to bind to FGF (fibroblast growth factor) receptors, similar to heparin, heparan sulfate, and sulfated alginate [35,36,37]. Further has it been shown that the binding and inhibition of chemo- and cytokines, such as transforming growth factor-β1 (TGF-β1), by sulfated fucans is size dependent. Even though the underlying mechanism is not yet resolved, a similar binding preference could play a role [38].

Koyanagi et al. demonstrated that highly sulfated fucans are able to bind VEGF [29].

In addition, it has been found that polymers with conjugated inhibitors have a higher inhibitory effect on tumor necrosis factor-α (TNF-α) if the inhibitors are attached to higher-molecular-weight polymers; this is due to an increased diffusion time [39]. Since the binding of VEGF to highly sulfated fucans has been proven, a similar effect of increased diffusion time could play a role.

FCSP are a diverse group of macromolecules with heterogeneous molecular structures. Still, due to its almost exclusive fucose sugar composition and the full sulfation of almost every sugar unit, LH sulfated fucan is rather homogeneous in nature, even when hydrolyzed to different molecular weight averages. Hence, it can be speculated that biological effects on cells and the metabolism are less likely caused by chemical reaction or direct receptor interactions only expressed by Fuc1 and not Fuc2 and Fuc3, but rather by physical and steric interactions such as stronger cell surface adhesion or stronger entanglement with proteins (cadherins or cytokines) due to the higher average molecular weight.

For further clarification, other FCSP with similar M_w_ but lower DS [14] should be included in future studies, as well as high-molecular-weight hyaluronic acids, alginates, and sulfated alginates [40,41] to better elucidate and distinguish the effects caused by molecular size, charge density, and degree of sulfation and to determine if branching points and sugar composition play an additional role. Potential FCSP–cytokine binding needs to be investigated to exclude potential masking of the determined VEGF secretion. Finally, a chromatographic fractionation of high-molecular-weight LH sulfated fucan should be applied to narrow down the molecular weight range and the polydispersity and to get access to LH sulfated fucan fractions of even higher molecular weight, potentially amplifying the previously expressed effects and directly relating them to specific molecular weight ranges.

From the results taken together, the sulfated fucan Fuc1 from LH with a molecular weight average of 1548 kDa seems to be best candidate for further research concerning AMD-relevant mechanisms. It did not lower the cell viability of primary RPE cells, and 50 µg/mL inhibited VEGF most efficiently. High molecular weight appears to be desirable for VEGF inhibition. We tested several sulfated fucans from different algal species previously; sulfated fucans from SL were also effective in oxidative stress protection in the OMM-1 and ARPE-19 cell lines, and 10 µg/mL SL sulfated fucan inhibited VEGF in ARPE-19 and RPE cells [11]. The molecular weight for this sulfated fucan was 1407 kDa (unpublished data), so it is comparable to Fuc1, which also lowered VEGF significantly at 10 µg/mL in RPE and ARPE-19. This renders SL and LH sulfated fucans as promising candidates for further AMD-related studies. Of note, the pure LH sulfated fucan in this study can be sustainably reproduced in high amounts for further experiments in perfusion cultures or in vivo studies. Furthermore, because of its protective effect in the sensitive OMM-1 cell line, it could be considered for future studies with neuronal model systems concerning oxidative stress protection. For further research, tests concerning inflammation, lipid metabolism, and complement systems would be of additional interest.

## 4. Material and Methods

### 4.1. Cell Culture

The uveal melanoma cell line OMM-1 [42] was provided by Dr. Sarah Coupland. RPMI 1640 (Merck, Darmstadt, Germany) was used for cultivation (with 10% fetal calf serum (Linaris GmbH, Wertheim-Bettingen, Germany) and 1% penicillin/streptomycin (Merck, Darmstadt, Germany)). 

The human RPE cell line ARPE-19 [43] was purchased from ATCC. The cultivation medium was HyClone DMEM (GE Healthcare, München, Germany) with 1% penicillin/streptomycin, 1.2% HEPES, 1% non-essential amino acids (all from Merck, Darmstadt, Germany), and 10% fetal calf serum, as previously described [11]. 

Primary porcine RPE cells were prepared as described before [30,44]. RPE cells were detached from porcine eyes by trypsin incubation and cultivated in HyClone DMEM (GE Healthcare, München, Germany) supplemented with penicillin/streptomycin (1%), HEPES (2.5%), non-essential amino acids (1%) (all Merck, Darmstadt, Germany), and 10% fetal calf serum. 

ARPE-19 and RPE were treated at confluence, and OMM-1 cells were treated at subconfluence.

### 4.2. Sulfated Fucan

Extraction: Freshly frozen LH was thawed, blended into small particles (≤ 3 mm), and kept in 90 °C H_2_O for 6 h. The solution was filtered (20–25 µm particle retention) using a vacuum pump; CaCl_2_ was added to the filtrate and it was filtrated again (5 µm particle retention). The newly obtained filtrate was purified through ultrafiltration (300 kDa MWCO) and lyophilized. 

Hydrolyzation: Lower-molecular-weight samples were obtained by mild acid hydrolysis of the ultrapurified sulfated fucan at pH = 3.0, 70 °C, and 5 min for Fuc2 and 35 min for Fuc3. The hydrolysates were cooled in ice water, dialyzed again with distilled water for 3 h (12 kDa MWCO), and lyophilized.

The purified and dried LH sulfated fucans Fuc1, Fuc2, and Fuc3 were dissolved in 5 mg/mL Ampuwa bidest (Fresenius, Schweinfurt, Germany). Before testing, the extracts were filtered with 0.2 µm Sarstedt filters (Nümbrecht, Germany) and further diluted in adequate medium to the concentrations mentioned in 4.6, 4.7, 4.8 and 4.9.

### 4.3. Monosaccharide Analysis

The sulfated fucan samples were hydrolyzed using 3 mol/L trifluoroacetic acid (TFA) at 95 °C for 12 h. The hydrolyzed monosaccharides were fractionated and analyzed through ion-exchange chromatography equipped with a CarboPac PA20 (Thermo Fisher Scientific Inc., Waltham, MA, USA) column.

### 4.4. Sulfate Content

The sulfate content of the ultrapurified sulfated fucan Fuc1 was externally analyzed using an Agilent 7500 Series quadrupole ICP-MS. The sulfate content of the hydrolyzed sulfated fucans was analyzed by Raman spectroscopy as previously described [12]. Raman spectra were recorded at room temperature using a Horiba Jobin–Yvon LabRAM HR system equipped with a Raman microscope (HORIBA Ltd., Kyoto, Japan).

### 4.5. Molecular Weight Determination

The molecular weight of the sulfated fucan was determined by size-exclusion chromatography (SEC) in the form of HPLC equipped with online multiangle static light scattering (MALS). The measurements were performed at ambient temperature using a Shodex LB-806 and a 2500 PWXL column as a separator. The measurement was executed with a Dawn HELEOS-8+ multiangle laser light scattering photometer (Wyatt, Santa Barbara, CA, USA) (λ_0_ = 660 nm) and a subsequent Optilab T-rEX differential refractometer. The mobile phase was 0.10 mol/L Na_2_HPO_4_ (pH = 7), and the flow rate was 0.2 mL/min. The injection volumes were 50 µL and 100 μL, with 1 g/mL. The data were obtained and processed using Astra (v. 7) software (Wyatt, Santa Barbara, CA, USA).

### 4.6. Oxidative Stress

To induce oxidative-stress-related cell death, OMM-1 cells were treated with 1 mM H_2_O_2_ and ARPE-19 cells were treated with 500 µM TBHP as previously established [11,16] to reduce cell viability to nearly 50% (tested via MTS assay, see below). Before insult with the oxidative agents, cells were incubated with 1, 10, 50, and 100 µg/mL LH extracts.

### 4.7. Methyl Thiazolyl Tetrazolium (MTT) Assay

The widely used MTT assay [45] was conducted as previously described [4,11] and was used after taking supernatants for the VEGF ELISA (see below) and after 24 h sulfated fucan (1–100 µg/mL) stimulation. The cells were incubated with 0.5 mg/mL MTT for 2 h and dissolved in DMSO. Measurement was taken at 550 nm with an Elx800 (BioTek Instruments Inc., Bad Friedrichshall, Germany).

### 4.8. MTS Assay

The CellTiter 96^®^ AQueous One Solution Cell Proliferation Assay from Promega Corporation (Mannheim, Germany) was used to measure the cell viability after 24 h treatment with 1–100 µg/mL LH extracts and in parallel to determine any protective effects after insult with oxidative stress. The abovementioned media and supplements were used without phenol red. To each well, 20 µl MTS was added, and the plates were incubated for 1 h.

### 4.9. VEGF ELISA

ARPE-19 and RPE supernatants were collected after treatment with LH extracts (1, 10, 50, and 100 µg/mL) on Day 3 after stimulation [11]. Medium with sulfated fucan was exchanged 24 h (ARPE-19) or 4 h (RPE) before collecting supernatants. Human VEGF DuoSet^®^ ELISA was used for ARPE-19, whereas the Human VEGF Quantikine^®^ ELISA was used for RPE supernatants (both R&D Systems, Wiesbaden, Germany). ELISAs were performed according to the manufacturer’s instructions. Untreated cells and medium samples were tested as controls. To set the VEGF secretion in relation to the cell viability, MTT assay was conducted after collection of the supernatants on Day 3.

### 4.10. Statistics

Experiments were independently repeated at least three times. Statistics were calculated in Microsoft Excel (Excel 2010, Microsoft) and GraphPad PRISM 7 (GraphPad Software, Inc. 2017). *p* values of <0.05, calculated via ANOVA, were considered significant. The diagram bars and lines represent the mean and standard deviation, respectively.

## 5. Conclusions

The aim of this study was to investigate the effects of three pure sulfated fucans from *Laminaria hyperborea*, which differed in their molecular size. The sulfated fucan origin and extraction method were the same, and they differed only in the molecular weight average (Fuc1 > Fuc2 > Fuc3), paving the way for comparable data. To test whether they are suitable for further testing for potential treatment of, e.g., AMD, different tests regarding oxidative stress protection (MTS), cell viability (MTT/MTS), and VEGF interference (VEGF ELISA) in ocular cells (OMM-1, ARPE-19, and RPE) were performed. The cell viability of ARPE-19 and RPE was not influenced by up to three days of treatment; this is important for further studies because it excludes toxic effects. Fuc2 and Fuc3 lowered the cell viability of the melanoma cell line OMM-1 and should therefore be tested in further tumor cell lines as to whether this is cell line specific or in general for tumor cells, which could pave the way for anticancer studies. Fuc1, the biggest sulfated fucan, was the only one which showed protective effects against oxidative stress (in OMM-1). Fuc3 reduced VEGF secretion in ARPE19 but stimulated VEGF secretion in primary RPE cells. Conversely, Fuc2 and Fuc1 inhibited VEGF in ARPE-19 as well as in RPE, with the strongest effect seen for 50 µg/mL Fuc1. Altogether, the results showed that the biological activity of sulfated fucans is dependent on the molecular weight, and the desired effect for the treatment of ocular diseases increases with the size of the sulfated fucan.

## Figures and Tables

**Table 1:** Data overview of the sulfated fucan samples used in this study. Degree of sulfation (DS), weight average molar mass (M_w_), number average molar mass (M_n_), degree of polymerization (DP), Z-average radius of gyration (R_z_), refractive index increment (dn/dc), and slope of the RMS conformation plot (b = rms versus M).

	DS	M_w_ [kDa]	M_n_ [kDa]	DP_n_	R_z_ [nm]	dn/dc	b
Fuc1	1.7	1548	1021	3512	83.0	0.115	0.66
Fuc2	1.7	499	241	829	47.1	0.115	0.66
Fuc3	1.7	26.9	13.9	48	-	0.115	-

## References

[1] Deniaud-Bouët E., Hardouin K., Potin P., Kloareg B., Hervé C. (2017). A review about brown algal cell walls and fucose-containing sulfated polysaccharides: Cell wall context, biomedical properties and key research challenges. Carbohydr. Polym..

[2] Li B., Lu F., Wei X., Zhao R. (2008). Fucoidan: Structure and bioactivity. Molecules.

[3] Klettner A. (2016). Fucoidan as a Potential Therapeutic for Major Blinding Diseases—A Hypothesis. Mar. Drugs.

[4] Dithmer M., Fuchs S., Shi Y., Schmidt H., Richert E., Roider J., Klettner A. (2014). Fucoidan reduces secretion and expression of vascular endothelial growth factor in the retinal pigment epithelium and reduces angiogenesis in vitro. PLoS ONE.

[5] Miller J.W. (2013). Age-related macular degeneration revisited—piecing the puzzle: The LXIX Edward Jackson memorial lecture. Am. J. Ophthalmol..

[6] Ding X., Patel M., Chan C.-C. (2009). Molecular pathology of age-related macular degeneration. Prog. Retin. Eye Res..

[7] Klettner A. (2012). Oxidative stress induced cellular signaling in RPE cells. Front. Biosci..

[8] Hageman G.S., Anderson D.H., Johnson L.V., Hancox L.S., Taiber A.J., Hardisty L.I., Hageman J.L., Stockman H.A., Borchardt J.D., Gehrs K.M. (2005). A common haplotype in the complement regulatory gene factor H (HF1/CFH) predisposes individuals to age-related macular degeneration. Proc. Natl. Acad. Sci. USA.

[9] McHarg S., Clark S.J., Day A.J., Bishop P.N. (2015). Age-related macular degeneration and the role of the complement system. Mol. Immunol..

[10] Schmidt-Erfurth U., Chong V., Loewenstein A., Larsen M., Souied E., Schlingemann R., Eldem B., Monés J., Richard G., Bandello F. (2014). Guidelines for the management of neovascular age-related macular degeneration by the European Society of Retina Specialists (EURETINA). Br. J. Ophthalmol..

[11] Dörschmann P., Bittkau K.S., Neupane S., Roider J., Alban S., Klettner A. (2019). Effects of fucoidans from five different brown algae on oxidative stress and VEGF interference in ocular cells. Mar. Drugs.

[12] Kopplin G., Rokstad A.M., Mélida H., Bulone V., Skjåk-Bræk G., Aachmann F.L. (2018). Structural Characterization of Fucoidan from Laminaria hyperborea: Assessment of Coagulation and Inflammatory Properties and Their Structure–Function Relationship. ACS Appl. Bio Mater..

[13] Stefaniak–Vidarsson M.M., Gudjónsdóttir M., Marteinsdottir G., Sigurjonsson O.E., Kristbergsson K. (2017). Evaluation of bioactivity of fucoidan from laminaria with in vitro human cell cultures (THP-1). Funct. Foods Health Dis..

[14] Fitton J.H., Stringer D.N., Karpiniec S.S. (2015). Therapies from Fucoidan: An Update. Mar. Drugs.

[15] Smidsrød O., Moe S. (2008). Biopolymer Chemistry.

[16] Dithmer M., Kirsch A.-M., Richert E., Fuchs S., Wang F., Schmidt H., Coupland S.E., Roider J., Klettner A. (2017). Fucoidan Does Not Exert Anti-Tumorigenic Effects on Uveal Melanoma Cell Lines. Mar. Drugs.

[17] Karlsson M., Kurz T. (2016). Attenuation of iron-binding proteins in ARPE-19 cells reduces their resistance to oxidative stress. Acta Ophthalmol..

[18] Bittkau K.S., Dörschmann P., Blümel M., Tasdemir D., Roider J., Klettner A., Alban S. (2019). Comparison of the Effects of Fucoidans on the Cell Viability of Tumor and Non-Tumor Cell Lines. Mar. Drugs.

[19] Banafa A.M., Roshan S., Liu Y.-Y., Chen H.-J., Chen M.-J., Yang G.-X., He G.-Y. (2013). Fucoidan induces G1 phase arrest and apoptosis through caspases-dependent pathway and ROS induction in human breast cancer MCF-7 cells. J. Huazhong Univ. Sci. Technol..

[20] Liu J.M., Bignon J., Haroun-Bouhedia F., Bittoun P., Vassy J., Fermandjian S., Wdzieczak-Bakala J., Boisson-Vidal C. (2005). Inhibitory Effect of Fucoidan on the Adhesion of Adenocarcinoma Cells to Fibronectin. Anticancer Res..

[21] Borazjani N.J., Tabarsa M., You S., Rezaei M. (2017). Improved immunomodulatory and antioxidant properties of unrefined fucoidans from Sargassum angustifolium by hydrolysis. J. Food Sci. Technol..

[22] Blasi M.A., Maresca V., Roccella M., Roccella F., Sansolini T., Grammatico P., Balestrazzi E., Picardo M. (1999). Antioxidant pattern in uveal melanocytes and melanoma cell cultures. Investig. Ophthalmol. Vis. Sci..

[23] Foresti R., Bucolo C., Platania C.M.B., Drago F., Dubois-Randé J.-L., Motterlini R. (2015). Nrf2 activators modulate oxidative stress responses and bioenergetic profiles of human retinal epithelial cells cultured in normal or high glucose conditions. Pharmacol. Res..

[24] Ryu M.J., Chung H.S. (2016). Fucoidan reduces oxidative stress by regulating the gene expression of HO-1 and SOD-1 through the Nrf2/ERK signaling pathway in HaCaT cells. Mol. Med. Rep..

[25] Vomund S., Schäfer A., Parnham M.J., Brüne B., von Knethen A. (2017). Nrf2, the Master Regulator of Anti-Oxidative Responses. Int. J. Mol. Sci..

[26] Wang Y.-Q., Wei J.-G., Tu M.-J., Gu J.-G., Zhang W. (2018). Fucoidan Alleviates Acetaminophen-Induced Hepatotoxicity via Oxidative Stress Inhibition and Nrf2 Translocation. Int. J. Mol. Sci..

[27] Marinval N., Saboural P., Haddad O., Maire M., Bassand K., Geinguenaud F., Djaker N., Ben Akrout K., La Lamy de Chapelle M., Robert R. (2016). Identification of a Pro-Angiogenic Potential and Cellular Uptake Mechanism of a LMW Highly Sulfated Fraction of Fucoidan from Ascophyllum nodosum. Mar. Drugs.

[28] Chen H., Cong Q., Du Z., Liao W., Zhang L., Yao Y., Ding K. (2016). Sulfated fucoidan FP08S2 inhibits lung cancer cell growth in vivo by disrupting angiogenesis via targeting VEGFR2/VEGF and blocking VEGFR2/Erk/VEGF signaling. Cancer Lett..

[29] Koyanagi S., Tanigawa N., Nakagawa H., Soeda S., Shimeno H. (2003). Oversulfation of fucoidan enhances its anti-angiogenic and antitumor activities. Biochem. Pharmacol..

[30] Klettner A., Roider J. (2008). Comparison of bevacizumab, ranibizumab, and pegaptanib in vitro: Efficiency and possible additional pathways. Investig. Ophthalmol. Vis. Sci..

[31] Klettner A., Westhues D., Lassen J., Bartsch S., Roider J. (2013). Regulation of constitutive vascular endothelial growth factor secretion in retinal pigment epithelium/choroid organ cultures, P38, nuclear factor κB, and the vascular endothelial growth factor receptor-2/phosphatidylinositol 3 kinase pathway. Mol. Vis..

[32] Liu F., Wang J., Chang A.K., Liu B., Yang L., Li Q., Wang P., Zou X. (2012). Fucoidan extract derived from Undaria pinnatifida inhibits angiogenesis by human umbilical vein endothelial cells. Phytomedicine.

[33] Narazaki M., Segarra M., Tosato G. (2008). Sulfated polysaccharides identified as inducers of neuropilin-1 internalization and functional inhibition of VEGF165 and semaphorin3A. Blood.

[34] Lake A.C., Vassy R., Di Benedetto M., Lavigne D., Le Visage C., Perret G.Y., Letourneur D. (2006). Low molecular weight fucoidan increases VEGF165-induced endothelial cell migration by enhancing VEGF165 binding to VEGFR-2 and NRP1. J. Biol. Chem..

[35] Foxall C., Wei Z., Schaefer M.E., Casabonne M., Fugedi P., Peto C., Castellot J.J., Brandley B.K. (1996). Sulfated malto-oligosaccharides bind to basic FGF, inhibit endothelial cell proliferation, and disrupt endothelial cell tube formation. J. Cell. Physiol..

[36] Kwak J.-Y. (2014). Fucoidan as a marine anticancer agent in preclinical development. Mar. Drugs.

[37] Arlov Ø., Aachmann F.L., Feyzi E., Sundan A., Skjåk-Bræk G. (2015). The Impact of Chain Length and Flexibility in the Interaction between Sulfated Alginates and HGF and FGF-2. Biomacromolecules.

[38] Kim T.H., Lee E.K., Lee M.J., Kim J.H., Yang W.S. (2013). Fucoidan inhibits activation and receptor binding of transforming growth factor-β1. Biochem. Biophys. Res. Commun..

[39] Washburn N.R., Prata J.E., Friedrich E.E., Ramadan M.H., Elder A.N., Sun L.T. (2013). Polymer-conjugated inhibitors of tumor necrosis factor-α for local control of inflammation. Biomatter.

[40] Rayahin J.E., Buhrman J.S., Zhang Y., Koh T.J., Gemeinhart R.A. (2015). High and low molecular weight hyaluronic acid differentially influence macrophage activation. ACS Biomater. Sci. Eng..

[41] Arlov Ø., Skjåk-Bræk G., Rokstad A.M. (2016). Sulfated alginate microspheres associate with factor H and dampen the inflammatory cytokine response. Acta Biomater..

[42] Luyten G.P., Naus N.C., Mooy C.M., Hagemeijer A., Kan-Mitchell J., van Drunen E., Vuzevski V., de Jong P.T., Luider T.M. (1996). Establishment and characterization of primary and metastatic uveal melanoma cell lines. Int. J. Cancer.

[43] Dunn K.C., Aotaki-Keen A.E., Putkey F.R., Hjelmeland L.M. (1996). ARPE-19, a human retinal pigment epithelial cell line with differentiated properties. Exp. Eye Res..

[44] Wiencke A.K., Kiilgaard J.F., Nicolini J., Bundgaard M., Röpke C., La Cour M. (2003). Growth of cultured porcine retinal pigment epithelial cells. Acta Ophthalmol. Scand..

[45] Riss T.L., Moravec R.A., Niles A.L., Duellman S., Benink H.A., Worzella T.J., Minor L., Sittampalam G.S., Coussens N.P., Brimacombe K., Grossman A., Arkin M., Auld D., Austin C., Baell J., Bejcek B., Caaveiro J.M.M. (2016). Cell Viability Assays. Assay Guidance Manual [Internet].

